# Cutaneous Manifestation of Acute Lymphoblastic Leukemia

**DOI:** 10.7759/cureus.74022

**Published:** 2024-11-19

**Authors:** Paula Cerqueira, Sara Pereira, Raquel Costa, Ana Garrido Gomes

**Affiliations:** 1 Internal Medicine, Unidade Local de Saúde do Alto Minho - Hospital Conde de Bertiandos, Ponte de Lima, PRT; 2 Internal Medicine, Hospital Distrital de Santarém, Santarém, PRT

**Keywords:** acute lymphoblastic leukemia, chemotherapy resistance, complexity of care, cutaneous manifestations, skin lesions differential diagnosis

## Abstract

Acute lymphoblastic leukemia (ALL) is a hematological neoplasm characterized by the proliferation of immature lymphoblasts. Although it is more common in children, it can also occur in adults, presenting significant clinical challenges. This case describes a 46-year-old male patient with a history of hypertension and dyslipidemia, whose first clinical manifestation of ALL was cutaneous. The patient showed resistance to initial chemotherapy, leading to complex management and, unfortunately, to a bad outcome.

## Introduction

Acute lymphoblastic leukemia (ALL) is a hematological neoplasm characterized by the uncontrolled proliferation of lymphoblasts, precursor cells of lymphocytes [1]. Although ALL is more prevalent in children, its presentation in adults has become increasingly recognized [2]. ALL is divided into two main subtypes: pre-B ALL and pre-T ALL, with the pre-B form being the most common among adults. Early diagnosis is crucial, as the disease can progress rapidly and lead to severe complications. The clinical manifestations of ALL are varied, with symptoms such as fever, fatigue, bone pain, and, in some cases, cutaneous involvement [2]. Although cutaneous manifestations are rare, they may include erythema, ecchymosis, and petechiae, reflecting leukemic cell infiltration or associated thrombocytopenia [3]. The standard treatment for ALL includes chemotherapy; however, treatment resistance is a common phenomenon, especially in adult patients who may present with more aggressive biological characteristics [4].

## Case presentation

A 46-year-old male patient presented in the emergency room with skin lesions that had developed over the last month and aggravated in the past two days. He reported that the lesions started as erythema non-pruritic but rapidly evolved into ecchymosis and petechiae, mainly on the legs, accompanied by fatigue and intermittent fever. He denied any history of recent trauma, as well as insect bites or previous ulcers. He had a medical history of controlled hypertension and dyslipidemia treated with statins and had no history or risk factors for immunosuppression.

**Figure 1:**
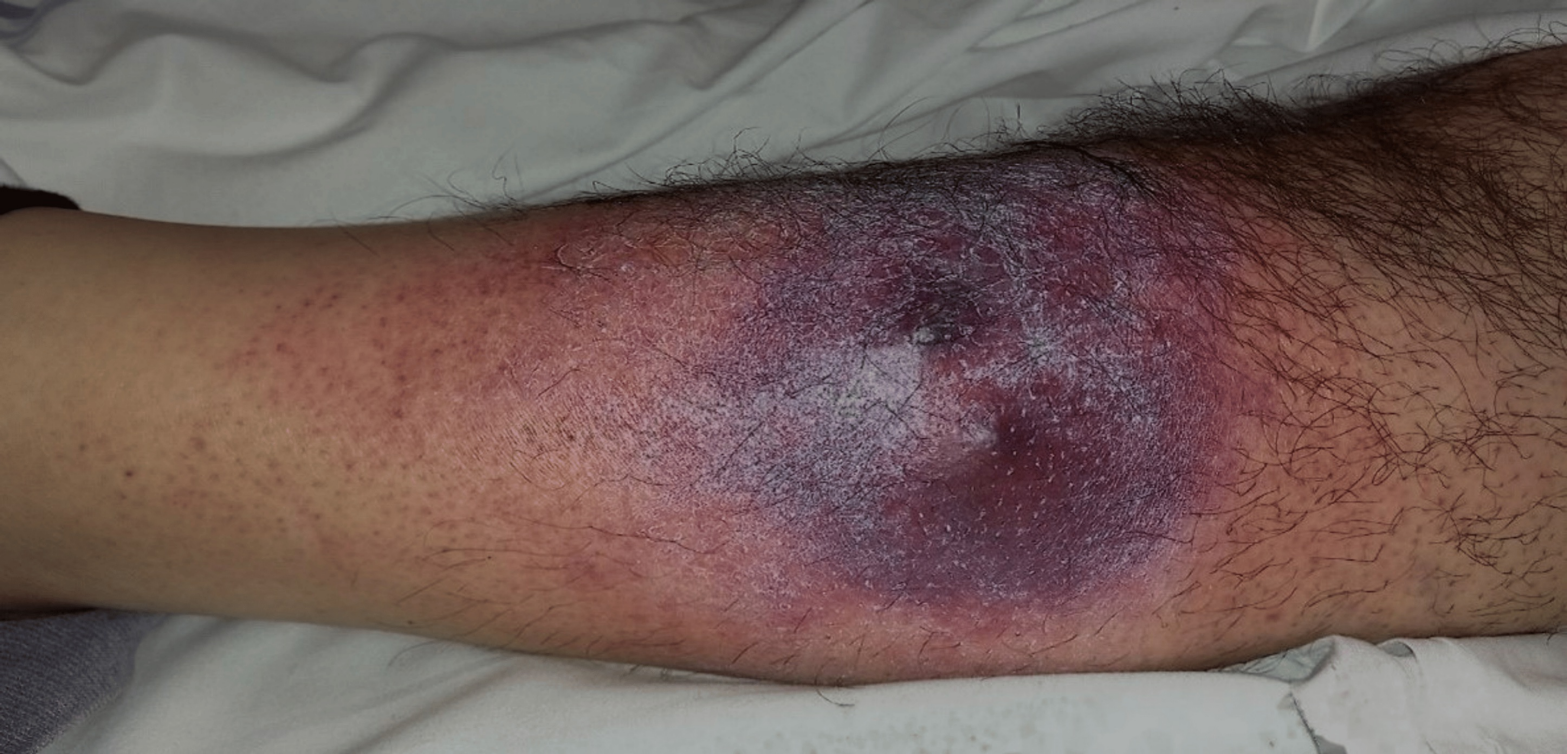
Indurated plaque on the left leg with erythema surrounding the lesion and petechiae.

On cutaneous examination, he had an erythematous-purplish single nodule surrounded by an indurated plaque on the left leg. He also had erythema surrounding the lesion and petechiae on the same leg (Figure 1).

The abdomen showed no palpable hepatomegaly or splenomegaly. A palpable, small, and painless enlarging left inguinal lymphadenopathy was observed. Based on the clinical presentation, initial laboratory tests were performed. The blood count revealed moderate anemia with hemoglobin at 9 g/dL, significant thrombocytopenia with a platelet count of 35,000/mm^3^, and leukocytosis with a count of 40,000/mm^3^, predominantly lymphoblasts. Blood tests also revealed impaired renal function, elevated CRP (14.64 mg/L), and elevated sedimentation rate (83 mm/hour).

During the patient's stay in the emergency room, he became progressively more hypotensive and tachycardic, with an increase in the respiratory rate. Given these lesions' new and quick progression and clinical worsening, the medical team initially considered a possible case of skin infection, vasculitis, or hematologic disorder. The patient was treated with antibiotics, as it was believed the cause might be bacterial. A skin biopsy was also performed in the emergency room. However, despite the treatment, the lesions continued to spread, the patient's fatigue worsened, and there was doubt regarding the initial hypothesis of an isolated skin infection.

A blood smear confirmed the presence of lymphoblasts, suggesting a diagnosis of leukemia. To confirm the diagnosis, a bone marrow aspiration was performed, revealing hypercellularity with lymphoblast infiltration, consistent with ALL. Flow cytometry identified the expression of typical B-cell markers supporting the disease subtype. The skin biopsy also showed medium-sized atypical cells with round to oval contours, scant cytoplasm, and finely dispersed chromatin infiltrating the dermis and subcutis. Since this finding is not pathognomonic, immunohistochemical staining and history remained crucial to distinguishing reactive from neoplastic infiltrates.

After the diagnosis, the patient started on induction chemotherapy, following a standard ALL protocol, a five-drug remission induction regimen with daunorubicin, vincristine, prednisone, pegaspargase, and cyclophosphamide. However, after two weeks of treatment, he had no significant clinical response and blood cell counts remained low, with persistent lymphoblasts in the bone marrow, indicating that the disease continued to progress despite therapeutic interventions. The patient began to experience complications in the same period, such as recurrent respiratory infections due to immunosuppression, despite receiving prophylactic therapy. Given the lack of response to treatment, additional tests were conducted, including genetic analysis, which revealed the presence of mutations associated with a high-risk profile (Philadelphia chromosome-positive). Given the resistance to chemotherapy and the progression of the disease, the medical team began discussions about integrating palliative care into the patient treatment approach. Unfortunately, the patient's clinical condition worsened, and he experienced a rapid deterioration in his general health. After a period of palliative care, the patient passed away due to complications associated with ALL and treatment resistance.

## Discussion

This case presents a rapidly progressing dermatological condition initially suspected of localized skin infection but ultimately diagnosed as ALL [5,6]. At the onset, the lesions suggested a common dermatological infection; however, the absence of antibiotic improvement after 48 hours raised concerns about a more profound, systemic issue.

Additionally, worsening systemic symptoms have shifted the clinical focus from a localized infection to a more complex systemic disease. Detailed laboratory testing revealed hematological abnormalities, and a subsequent bone marrow biopsy confirmed the diagnosis.

This case highlights several critical clinical points. First, skin lesions, especially those that progress rapidly or do not respond to standard treatments, warrant a broader differential diagnosis. While infections are common causes of skin lesions, systemic diseases such as malignancies or autoimmune conditions should be considered when the clinical presentation does not align with typical infections. Second, systemic symptoms, such as fever and fatigue, should raise suspicion of a possible malignancy besides infection. Third, complete laboratory testing and histopathological evaluation are critical in cases of diagnostic uncertainty, particularly when initial treatments fail. Lastly, this case underscores the clinical imitation often seen in hematological disorders (Table 1) [7]. The initial presentation could have easily been mistaken for cellulitis or vasculitis, potentially delaying the correct diagnosis [8].

**Table 1:** Differential diagnosis of leukemia cutis.

Differential Diagnosis of Leukemia Cutis
Lymphoma and pseudolymphoma
Metastatic solid tumors
Pyoderma gangrenosum
Vasculitis
Drug reactions
Urticaria
Viral exanthem
Hidradenitis
Erythema multiforme
Erythema nodosum
Infections due to immune suppression: cellulitis, herpes zoster, herpes simplex
Graft vs. host disease

The patient's demise highlights the complexity of managing ALL in adults and the importance of integrating palliative care in cases of advanced disease [9].

## Conclusions

This case highlights the unique challenges of managing ALL in an adult patient, mainly when the disease manifests initially with cutaneous symptoms. Although ALL is more frequently diagnosed in children, adult presentations are often more complex, especially with high-risk genetic profiles that may not respond well to standard treatments. The progression of the patient's condition despite intensive chemotherapy underscores the importance of early recognition of treatment resistance and the need for alternative strategies, including targeted therapies or bone marrow transplantation when feasible.

In cases of advanced disease with limited treatment options, as illustrated here, the integration of palliative care proved essential in maintaining the patient's quality of life, offering symptom relief, and providing psychological support to the patient and family. This case reinforces the importance of a multidisciplinary approach in managing adult ALL, particularly for patients with poor prognoses, where holistic and compassionate care becomes paramount. Ultimately, the incorporation of palliative care allowed for a dignified end-of-life experience, demonstrating its critical role in the comprehensive treatment of hematologic malignancies.
